# Single-cell RNA-seq analysis of longitudinal CD4^+^ T cell samples reveals cell-type-specific changes during early stages of type 1 diabetes

**DOI:** 10.1186/s13073-025-01574-x

**Published:** 2025-12-29

**Authors:** Rahul Biradar, Ubaid Ullah Kalim, Tapio Lönnberg, Sini Junttila, Tomi Suomi, Sebastián Zúñiga Norman, Inna Starskaia, Niklas Paulin, Lea Mikkola, Outi Vaarala, Omid Rasool, Mikael Knip, Laura L. Elo, Riitta Lahesmaa

**Affiliations:** 1 Turku Bioscience Centre, University of Turku and Åbo Akademi University, Turku, Finland; 2 InFLAMES Research Flagship Center, University of Turku, Turku, Finland; 3 Orion Pharma, Espoo, Finland; 4 Research Program for Clinical and Molecular Metabolism, Faculty of Medicine, University of Helsinki, Helsinki, Finland; 5 Tampere Centre for Child Health Research, Tampere University Hospital, Tampere, Finland; 6 Paediatric Research Centre, Helsinki University Hospital, Helsinki, Finland; 7 Institute of Biomedicine, University of Turku, Turku, Finland

**Keywords:** Type 1 diabetes (T1D), Seroconversion, CD4^+^ T cells, Single-cell RNA sequencing scRNA-seq, Regulons

## Abstract

**Background:**

T cells play a pivotal role in the autoimmune destruction of beta cells in type 1 diabetes. However, our understanding of the disease has been limited by lack of a comprehensive single-cell transcriptome analysis of T cells during its early stages.

**Methods:**

We performed single cell RNA sequencing analysis of 73 longitudinal CD4^+^ T cell samples collected at an early age of 3–24 months from children who subsequently developed type 1 diabetes (*N* = 11) and their matched controls (*N* = 11). The samples analysed here were at or before the age of seroconversion, i.e., appearance of beta cell specific autoantibodies. These samples were obtained from the Trial to Reduce Insulin Dependent Diabetes Mellitus (IDDM) in Genetically at Risk (TRIGR) study (ClincalTrials.gov ID: NCT00179777).

**Results:**

By phenotypically characterizing over 99,000 cells, we identified cell-type-specific gene expression patterns associated with disease progression. While the cell-type compositions were similar, several genes were differentially regulated in cases in different cell types. Besides pathways altered in cases in specific cell types, interferon related pathways and pathways related to viral response were altered in multiple cell types in cases. We also identified gene regulatory networks (regulon) that drives the transcriptional state of the cell types. Notably, we observed increased *PRDM1* regulon activity in Th17 cells and diminished *GATA3* regulon activity in naïve T cells, among other changes in the activity of different regulons in children progressing to disease.

**Conclusions:**

Our findings reveal early, cell-type-specific changes in transcription and gene regulatory networks in CD4⁺ T cells associated with type 1 diabetes progression, highlighting key pathways and transcriptional regulators. These insights provide a foundation for understanding early immune dysregulation in type 1 diabetes and basis for strategies to develop early diagnosis and intervention**.**

**Graphical Abstract:**

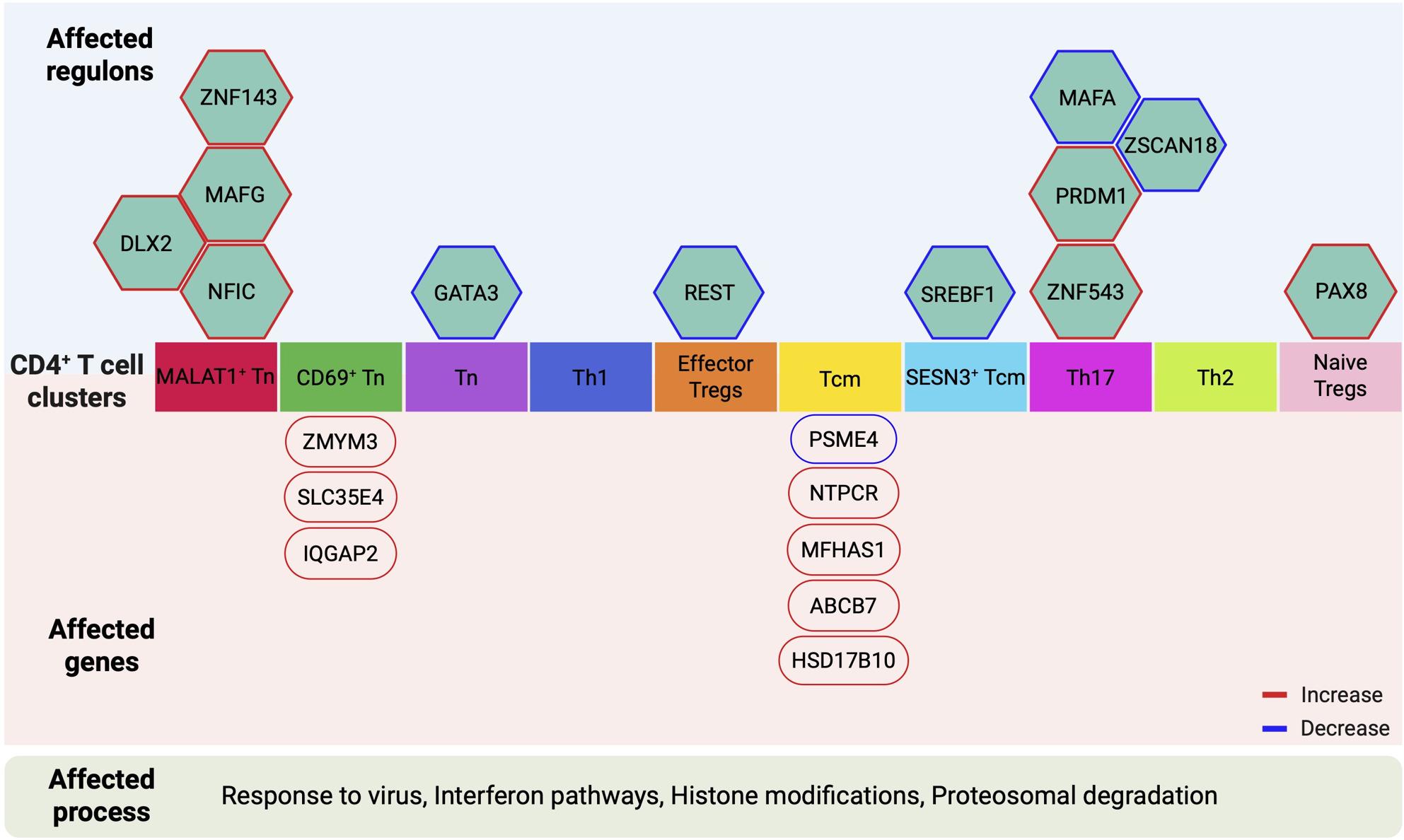

**Supplementary Information:**

The online version contains supplementary material available at 10.1186/s13073-025-01574-x.

## Background

Type 1 diabetes is a complex autoimmune disorder, characterized by the gradual loss of insulin-producing pancreatic beta cells, primarily driven by an autoimmune attack, in which T cells play a pivotal role. Although genome-wide association studies (GWAS) and immunological profiling have identified key genetic and environmental contributors, the early immunological events that precede clinical onset remain incompletely understood. The emergence of circulating autoantibodies associated with type 1 diabetes (i.e., seroconversion) is the earliest indicator of an ongoing disease process. However, the presence of autoantibodies reflects an already active immune response and does not provide insight into the molecular and cellular events that initiate disease development. While most children who develop multiple autoantibodies will eventually progress to clinical disease, the precise immunological and cellular mechanisms that precede seroconversion remain poorly understood [1, 2].

We and others showed that immune activity is detected in peripheral blood before seroconversion [3–5]. Previous studies of prospective samples preceding seroconversion primarily examined whole blood or peripheral blood mononuclear cell (PBMC) samples by bulk approaches [6, 7]. However, transcriptome studies with bulk RNA sequencing provide only an average profile of all cell types in the sample. This limitation obscures cell-specific gene expression patterns and underlines the need for single-cell RNA sequencing (scRNA-seq) analysis. Earlier scRNA-seq studies on the other hand analyzed samples post disease onset [8, 9].

To address this gap, we performed longitudinal scRNA-seq of CD4⁺ T cells isolated from PBMCs of children genetically at-risk of developing type 1 diabetes. We compared 11 children who later progressed to beta cell autoimmunity and clinical disease to their respective controls who were matched for HLA-risk, age, and sex but did not progress to beta-cell autoimmunity during the follow-up of up to 10–15 years of age. We focused on CD4^+^ T cells, given their central role in orchestrating immune responses. Moreover, a recent study investigating gene expression in CD4^+^ and CD8^+^ T cells, natural killer (NK) cells, and B cells using RNA-seq found that gene expression differences in children progressing to type 1 diabetes were largely confined to CD4^+^ T cells [6]. Importantly, type 1 diabetes exhibits significant heterogeneity in the rate of disease progression, with age of onset ranging from infancy to adulthood [10]. In this study, we analysed samples from a homogenous cohort of children who progressed to clinical disease at an early age (below 5 years).

Our study provides the first comprehensive single-cell analysis of CD4⁺ T cell transcriptome during the earliest phases of type 1 diabetes development—prior to both seroconversion and clinical diagnosis. Through this approach, we identify distinct transcriptional signatures, perturbed gene regulatory networks, and altered interferon and viral response pathways that may contribute to the pathogenesis of type 1 diabetes. These findings offer new insights into the temporal and cell-type–specific immune mechanisms driving progression to autoimmunity in early-onset type 1 diabetes.

## Methods

### Study cohort

Samples were obtained from the Trial to Reduce Insulin Dependent Diabetes Mellitus (IDDM) in Genetically at Risk (TRIGR) study, an international, multicenter, double-blind randomized clinical trial conducted between 2002 and 2017 (ClinicalTrials.gov ID: NCT00179777) [11]. The TRIGR study population includes new-born infants who had at least one family member affected by type 1 diabetes and had one of the following HLA genotypes: (a) HLA-DQB1*0302/DQA1*05-DQB1*02; (b) HLA-DQB1*0302/x (x not DQB1*02, DQB1*0301, or DQB1*0602); (c) HLA-DQA1*05-DQB1*02/y (y not DQA1*0201-DQB1*02, DQB1*0301, DQB1*0602, or DQB1*0603); (d) HLA-DQA1*03-DQB1*02/y (y not DQA1*0201-DQB1*02, DQB1*0301, DQB1*0602, or DQB1*0603). Other inclusion and exclusion criteria for the TRIGR study have been described earlier [11]. Participants were followed prospectively from birth up to 10–15 years of age. Type 1 diabetes was diagnosed according to WHO criteria [12]. Islet cell antibodies (ICA) were detected by indirect immunofluorescence in Oulu, Finland, with sensitivity and specificity of 100% and 98%, respectively [13]. Additional autoantibodies (IAA, GADA, IA-2A, ZnT8A) were analyzed in Helsinki, Finland, using radiobinding immunoassays with established disease-specific sensitivities and specificities [14, 15]. The TRIGR study was approved by the ethics committees of all participating centers, with written informed consent from parents. Children provided assent when appropriate for age according to local guidelines. The outcome of the trial was reported elswhere [16].

From TRIGR cohort, we selected a set of 11 genetically at-risk children who progressed rapidly to type 1 diabetes (cases) and their HLA-, age-, and sex-matched children who remained autoantibody negative and did not progress to the disease (controls). Case children seroconverted to one or more diabetes-associated autoantibodies by the age of 2 years and were diagnosed with type 1 diabetes between 1.6 and 4.3 years (mean 2.5 years), representing fast progressors. Clinical details of the case cohort are summarized in Table 1 and the sample metadata is provided as Additional file 2: Table S1. Across the 22 children (11 pairs), a total of 73 longitudinal PBMC samples were collected at 3, 6, 9, 12, 18, and 24 months of age. Case children were sampled until seroconversion and diagnosis, while matched controls were sampled in parallel. PBMCs were processed at TRIGR study sites, cryopreserved at –150 °C, and shipped to our laboratory to analyze using single-cell RNA sequencing.

**Table 1:** Table describing the type 1 diabetes cases of the cohort. The controls were matched for age, sex, and HLA risk group

Case ID	Sex	Age at Seroconversion (y)	First autoantibodies	Age at T1D diagnosis (y)
Case 1	Male	1.5	ZnT8A, ICA	3.9
Case 2	Male	0.9	ZnT8A, ICA	1.9
Case 3	Female	1.4	ZnT8A, ICA	4.3
Case 4	Male	0.5	IAA	1.8
Case 5	Male	2	IAA, GADA, ZnT8A, ICA	2.9
Case 6	Male	1.5	IAA, IA-2A, ZnT8A, ICA	2.4
Case 7	Male	1.5	IAA, ICA	2.9
Case 8	Male	1	IAA, IA-2A, ICA	2
Case 9	Male	0.75	IAA	1.6
Case 10	Male	0.75	IAA, ICA	2.5
Case 11	Female	1	GADA, IA-2A, ICA	1.6

### CD4^+^ T cell isolation from peripheral blood mononuclear cells

PBMC samples stored at −150 °C were thawed quickly in a 37 °C water bath and quantitated for cell numbers and viability using trypan blue staining with a Countess cell counter (ThermoFischer Scientific). On average, 94% of cells were viable. Dynabeads™ CD4^+^-positive isolation kit (Invitrogen #11331D) was used to enrich CD4^+^ T cells.

### Single-cell RNA-sequencing with cell multiplexing

Chromium Single Cell Kits (10X Genomics Single cell 3’ Reagent Kit V3.1) were utilized to analyze transcriptome of CD4^+^ T cells of children who rapidly progressed to type 1 diabetes and their controls. Super-loading of CD4^+^ T cells was achieved for every Chromium run with cell multiplexing using human cell hashing antibodies from BioLegend (Additional file 2: Table S2) as described before [17]. Briefly, up to 10 case and control CD4^+^ T cell samples were processed for cell multiplexing as described in Additional file 2: Table S3. All the samples from each pair (i.e., matched case and control child) were pooled as one multiplexed sample. A total of 0.5 million CD4^+^ T cells from each sample from every case and control child were independently stained with human cell hashtag antibody as per manufacturer’s protocol [17].

Stained cells were pooled together in equal proportions, i.e. 0.1 million cells from each sample, as one multiplexed sample and loaded onto Chromium Next GEM Chip G. For each run, we aimed at a recovery of 20,000 single cells by super-loading 33,000 cells at a cell concentration recommended by 10X Genomics. Cells in gel in emersion (GEM) were lysed for reverse transcription and complimentary DNA (cDNA) amplification. Full-length cDNA along with cell barcode identifiers were PCR-amplified. Two different cDNA sequencing libraries were prepared from reverse-transcribed polyadenylated transcripts of each cell and from cell hashtag oligo bound to the same cell, respectively, utilizing dual indexing. Sequencing of cDNA libraries was performed with Illumina NovaSeq 6000 at sequencing depth of 20,000 and 5,000 reads per cell for gene expression and cell hashtag libraries, respectively. The data were processed using Cell Ranger pipeline version 3.0.1. Computational demultiplexing of pooled samples was performed and single-cell transcriptome data were obtained.

### Analysis of scRNA-seq

Hashtag oligo demultiplexing and singlet identification were performed using HTODemux function in Seurat (v.4.0.1) in R (v.4.1.0) [18]. Only cells identified as singlets in the demultiplexing of hashtag oligos were retained in the analysis. Additionally, low-quality cells expressing less than 200 or more than 3,000 genes were filtered out. Cells expressing more than 3,000 genes were filtered out in order to remove potential doublets. High fraction of mitochondrial genes is indicative of cell damage [19, 20], therefore cells with more than 10% mitochondrial genes were also filtered out.

Log-transform normalization, identification of 2,000 most highly variable features, integration, scaling, dimensionality reduction with PCA and clustering were performed using Seurat (v.4.0.1) in R (v.4.1.0). The integration of all samples into one dataset was performed using Seurat’s reciprocal PCA method that identifies anchors between the integrated datasets [21]. Clustering used Seurat’s K-nearest neighbours graph-based method and Louvain algorithm. Clusters were visualised using Uniform Manifold Approximation and Projection (UMAP) and annotated automatically with Azimuth (v.0.3.2) [18] using a human PBMC reference. Cells annotated as monocytes, B cells or dendritic cells were removed from the analysis. After removal of the contaminating cell types, clustering was repeated with Seurat using a resolution value of 1.2, and the resulting clusters were annotated manually using conserved marker genes. Conserved marker genes were identified using Seurat’s FindConservedMarkers function. Pseudo-bulks for differential expression analysis were created by aggregating the counts across all cells in one sample in one cell type for each gene using R package muscat (v.1.6.0) [22].

### Longitudinal analysis using linear mixed effects modelling for cell type proportions and differentially expressed gene changes

To test the gene expression differences between cases and controls, linear mixed effect (LME) models were fitted for each gene, wherein disease status, age, their interaction, and sex were treated as fixed effects and the case–control pair was allowed random intercept [expression ~ status * age + sex + (1|pair)]. The 95% confidence intervals (from low/2.5% to high/97.5%) for the case–control coefficients were calculated. To test the cell type proportion differences between cases and control LME models were fitted for each cell, wherein disease status, age, their interaction, and sex were treated as fixed effects and the case–control pair was allowed random intercept [proportion ~ status * age + sex + (1|pair)]. The mixed effects modelling was implemented using lmerTest (v.3.1–3) R package. Correction for multiple testing was done using Benjamini–Hochberg adjustment. The analysis was performed separately for each cell type.

### Time point-wise differential expression analysis

R package ROTS (v.1.32.0) [23] was used to test for differences in expression between cases and controls at 3, 6, 9, 12 and 18 month time points. ROTS analysis was done on pseudo-bulk data where the gene expression from each different cell type cluster were combined to get the cell type-specific bulk expression of each gene in each sample. No additional variables were used in the analysis. The analysis was performed separately for each cell type.

### Pathway enrichment analysis

Gene set enrichment analysis (GSEA) for the ranked gene list of LME and ROTS analysis for each cell type, ClusterProfiler R package was used [24]. The gseGO function was applied on the LME coefficient ranked gene list from LME analysis or log2(foldchange) gene list from ROTS analysis for testing enrichment of gene ontology biological process (GOBP) terms, using following setting: OrgDb = org.Hs.eg.db, ont = “BP,” keyType = “SYMBOL,” pAdjustMethod = “BH,” qvalueCutoff = 0.05, exponent = 1, eps = 1e-300, nPerm = 10, 000, minGSSize = 10, and maxGSSize = 500. The *simplify* function was used to simplify the enriched terms. The top 15 significantly enriched terms (ranked by NES) and the cell type-specific terms of each cell type were selected for visualization. For over representation analysis on regulon-target genes, enrichGO function from ClusterProfiler R package was used. The enrichment of GOBP terms were tested using following setting: OrgDb = org.Hs.eg.db, ont = “BP,” keyType = “SYMBOL,” pAdjustMethod = “BH,” qvalueCutoff = 0.05, exponent = 1, eps = 1e-300, nPerm = 10, 000, minGSSize = 10, and maxGSSize = 500.

### TaqMan analysis

For validation, we selected the pre-seroconversion time point and used samples from five of 11 pairs (pairs: 2, 3, 6, 7, 10), based on availability. For RT-qPCR, RNA was isolated (AllPrep DNA/RNA/miRNA Universal kit, Cat# 80,224, QIAGEN) and treated in-column with DNase (RNase-Free Dnase Set, #79,254, QIAGEN) for 15 min. The removal of genomic DNA was ascertained by treating the samples with DNase I (Invitrogen, #18,068–015) before cDNA synthesis with SuperScript II Reverse Transcriptase (Invitrogen, #18,064,014). RT-qPCR was performed using KAPA Probe Fast Rox Low master mix (KAPA Biosystems, #kk4718) and amplification was monitored with QuantStudio 12 K Flex Real-Time PCR System (ThermoFisher Scientific). The Ct values were normalized against the signal acquired with EF1A. The following TaqMan assays and TaqMan primers and probe were used: IQGAP2 (ThermoFisher Scientific, Cat# 4,351,372, Assay ID: Hs01117929_m1), EF1A (Forward: 5'-CTGAACCATCCAGGCCAAAT-3', Reverse: 5'-GCCGTGTGGCAATCCAAT-3', Probe: 5'−6(FAM)-AGCGCCGGCTATGCCCCTG-(TAMRA)−3'.

### Regulon analysis

Transcription factors and their direct target genes, known as regulons, were predicted and analyzed using the SCENIC (v.0.12) method for network inference and motif discovery [25]. First, candidate regulatory modules were inferred from co-expression patterns between genes. Next, co-expression modules were refined by removing indirect targets using transcription factor motif information in a pre-calculated database provided with the SCENIC tool. The database used for this pruning step was the 500 bp upstream and 100 bp downstream of the transcription start site. Finally, the activity of the discovered regulons was measured in each cell as regulon specificity score and used for clustering. As an extension to the SCENIC results, the regulon activity was further binarized and contrasted with the scRNA-seq clustering results, where the activity of each regulon was plotted in the pre-calculated UMAP projection. A gene regulatory network map was generated using Cytoscape (v.3.9.1) [26] for those regulons showing statistically significant differences in their activity between the cases and controls using Wilcoxon signed-rank test. Regulons with P value < 0.01 were considered significant. Each transcription factor was assigned with at most 10 most important targets based on the SCENIC analysis.

### Statistical analyses

Statistical analyses were performed in R (v.4.1.0) unless otherwise stated. For linear mixed-effects models (gene expression and on cell-type proportions), p-values for fixed effects were calculated using Satterthwaite’s approximation as implemented in the lmerTest package, and multiple testing was controlled using the Benjamini–Hochberg false discovery rate (FDR) adjustment. Genes with FDR < 0.1 were considered significantly differentially expressed (DE) whereas *p* < 0.001 was used as cut off for identifying age-, or status-associated genes.

Time point–wise differential expression between cases and controls was assessed on pseudo-bulk data using reproducibility optimized test statistics (ROTS) (v.1.32.0). ROTS calculates *p*-values using permutation-based significance testing; genes with p-values < 0.001 were considered to be associated with case control status.

For TaqMan analysis, paired two-tailed students t-test was used to determine significance. The Gene set enrichment analyses were performed with clusterProfiler (gseGO/enrichGO) using pAdjustMethod = "BH" and a q-value cutoff of 0.05 (nPerm = 10,000, minGSSize = 10, maxGSSize = 500). Regulon activity differences from SCENIC (v.0.12) were tested using the paired Wilcoxon signed-rank test and regulons with nominal p < 0.01 were reported as significant. When applicable, the specific statistical method and multiple-testing correction are noted in the corresponding Methods subsection.

## Results

### Unsupervised clustering identified 10 clusters within CD4^+^ T cells

**Fig. 1:**
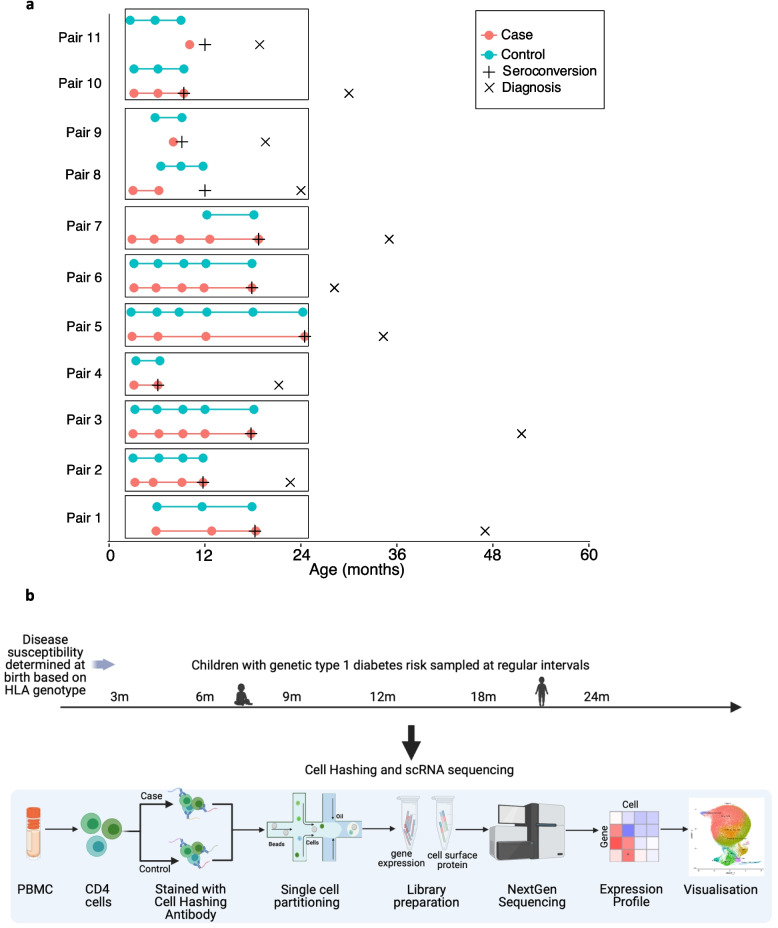
Scheme of sample collections and single cell RNA-seq. **a** Case child is visualized as a red line below the corresponding control child visualized as a cyan line. The sample collection time points are marked as circles. Seroconversion and diagnosis of type 1 diabetes are marked as + and x, respectively. Boxes around samples indicate sample pooling strategy, i.e., samples in the same box were pooled together (**b**) Outline of the sample collection from children with genetic type 1 diabetes risk sampled before seroconversion at regular intervals including 3 months, 6 months, 9 months, 12 months, 18 months, and 24 months and their respective sex and age matched controls. Experimental design of scRNA-seq experiment, where isolated CD4^+^ T cells from each pair of case and control child were processed together for cell multiplexing

To investigate early gene expression changes in CD4^+^ T cells during development of type 1 diabetes, we performed scRNA-seq of 73 longitudinal samples of CD4^+^ T cells isolated from PBMCs of 11 pairs of case and control children (Table 1). Samples were collected at 3, 6, 9, 12, 18, and 24 months of age. Case children were followed until seroconversion and diagnosis. All the children seroconverted by the age of 2 years and developed clinical disease by the age of 5 years (Fig. 1a).

To enable sample multiplexing, we used cell hashing antibodies (Fig. 1b; Additional file 2: Table S2–S3) and subsequently applied the HTODemux protocol to demultiplex pooled samples. After stringent quality control filtering to exclude low-quality cells and doublets (Additional file 1: Fig. S1), we retained 10,000–15,000 high-quality single cells per case–control pair, except for pairs 8–9 and 10–11, which yielded 7,112 and 8,888 cells, respectively (Additional file 1: Fig. S2a). The median gene count per cell was 1,178, ranging from 880 to 1,600 (Additional file 1: Fig. S2b).

**Fig. 2:**
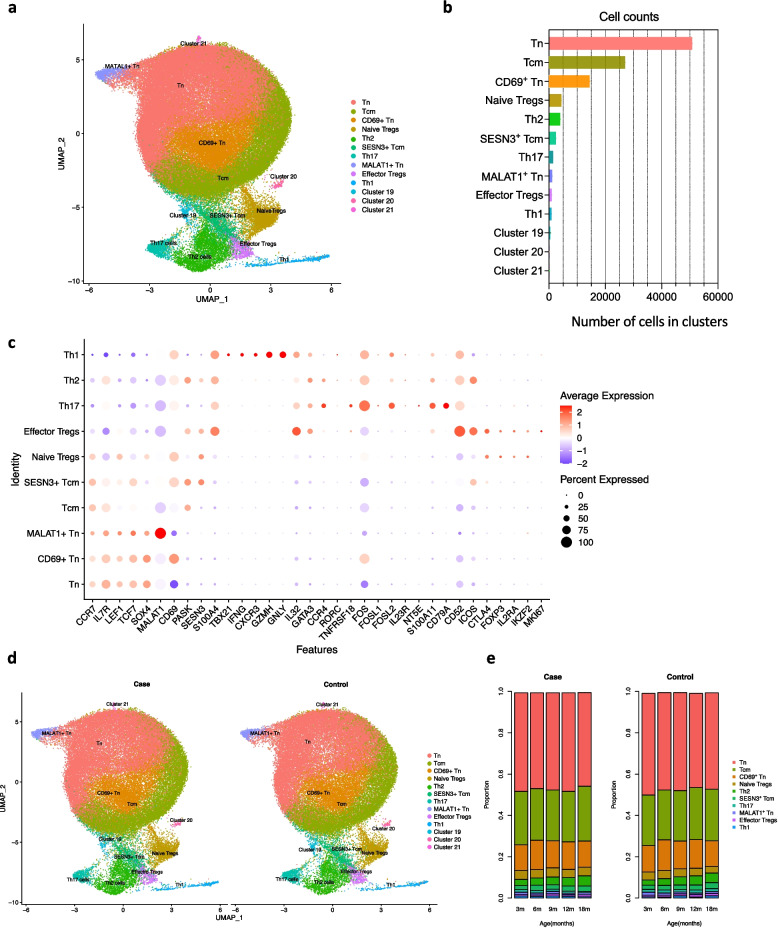
Single cell transcriptome profiling of children developing type 1 diabetes. **a** UMAP cell clusters from the pooled data from all the case and control children (in total 99,263 cells). Clusters are named according to the expression of marker genes for each subtype. **b** The figure shows the number of cells in each cell cluster, taking data of all case and control samples. **c** Dot plot displays the expression of marker genes in the 10 clusters. **d** UMAP indicating the identified CD4^+^ T cell subtypes in samples from case children and control children, separately. **e** Proportions of each CD4^+^ T cell subtype for case and control children during the first 18 months of age are calculated using LME modelling. Correction for multiple testing was done using Benjamini–Hochberg adjustment

All samples were combined for clustering, resulting in a dataset of 99,263 CD4⁺ T cells. We performed unsupervised clustering of the cells into 23 clusters (Additional file 1: Fig. S2c), followed by merging similar clusters based on expression of top marker genes (Additional file 2: Table S4). Clusters 1, 4, 5, 6, 8, 9, 10 and 11 were merged as naive T cells (Tn), clusters 0, 2, 7 and 22 merged as central memory T cells (Tcm) and clusters 3 and 9 were merged as CD69^+^ Tn cells (Additional file 1: Fig. S2c). After these steps, we obtained 13 clusters of CD4^+^ T cells (Fig. 2a). Clusters 19, 20 and 21 had fewer than 500 cells (Fig. 2b), and were excluded from further downstream analyses. The remaining 10 clusters contained cells from all samples and pooled batches, indicating that clustering was driven primarily by biological variation rather than batch effects (Additional file 1: Fig. S2d).

Among the 10 retained clusters, three had a naive phenotype, two displayed central memory characteristics, three were of effector phenotype, and two represented regulatory T cells (Tregs). The Tn cluster was defined by high expression of SOX4, as well as IL7R, LEF1, and TCF7 (Fig. 2c). Two additional clusters with naive phenotype were distinguished by elevated expression of *MALAT1* or *CD69*, and were thus named MALAT1⁺ Tn and CD69⁺ Tn, respectively. To assess whether CD69 expression in naive T cells was atypical, we examined a previously published paediatric PBMC scRNA-seq dataset (GEO accession: GSE206295) [27] and found that approximately half of the naive CD4^+^ T cells expressed CD69 (Additional file 1: Fig. S3). The Tcm cluster showed high expression of *PASK* and *CCR7* along with reduced *SOX4* expression. A second Tcm cluster expressing high levels of *SESN3* was designated as SESN3⁺ Tcm (Fig. 2c).

Of the effector clusters, the Th1 cluster expressed high levels of *TBX21*, *IFNG*, *CXCR3*, *GZMH*, and *GNLY*, and low levels of *CCR4*; the Th2 cluster expressed high *GATA3* and *CCR4*; and the Th17 cluster was marked by the expression *RORC*, *TNFRSF18*, *FOS*, *FOSL1*, *FOSL2*, *IL23R*, *NT5E*, *CD79A*, and *S100A11*. Both Tregs clusters expressed canonical markers *CTLA4*, *FOXP3*, *IL2RA*, and *IKZF2*. The effector Tregs subset additionally expressed *CD52*, *ICOS*, and *MKI67*, while the naive Tregs subset showed high *CCR7*, *LEF1*, and *SOX4* expression (Fig. 2c).

### Cell-type proportions were similar in cases and controls

To determine if there were differences in the cell-type proportions within the CD4^+^ T cell subtypes between the case and control children, we performed differential abundance analysis using linear mixed effects (LME) modelling. Each cluster was similarly represented by cells from case and control children (Fig. 2d-e), and no significant differences were observed (LME analysis, FDR < 0.1, multiple testing by *Benjamini–Hochberg method*). These results suggest that the relative abundance of CD4⁺ T cell subtypes remained unchanged between cases and controls.

### Differential gene expression analysis identified genes associated with disease status

**Fig. 3:**
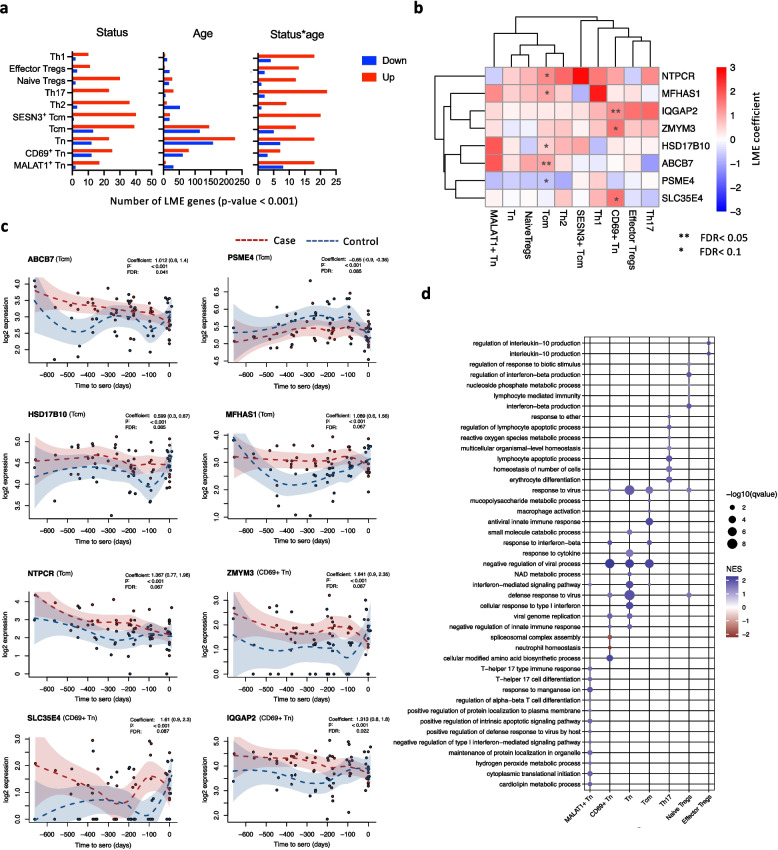
Gene expression changes between cases and controls. **a** Number of genes associated with case–control status (Status), age (Age), or their interaction Status*age), as identified by LME analysis (*p* < 0.001). Red and blue bars show the number of upregulated and downregulated genes in cases, respectively. **b** Heatmap shows the expression 8 DE genes between cases and controls in one or more subsets (FDR < 0.1). The color indicates the LME coefficient values, where red and blue indicate up and downregulation in cases as compared to controls, respectively. **c** The expression profiles of the 8 DE genes in the indicated cell type. The coefficient values include 95% confidence intervals, shown in brackets, (from low/2.5% to high/97.5%) while dashed LOESS lines with 95% confidence intervals are shown for mean expression in each group. The plots are seroconversion centered. Case and control samples have been indicated with red and blue, respectively. **d** The dot plot shows the enrichment of pathways in different cell types shown on x axis as obtained from GSEA

To identify genes associated with case–control status or age within each CD4^+^ T cell subtype, we performed differential gene expression analysis using LME modelling, accounting for the longitudinal design of the study (Methods). The model also included an interaction term between case–control status and age to detect genes with age-dependent expression patterns that differ between cases and controls. The analysis identified 285, 785, and 172 genes associated with status, age, or the status-age interaction term, respectively (*p* < 0.001) (Fig. 3a**;** Additional file 2: Table S5-S14). Interestingly, age-associated genes were predominantly identified in the Tn and Tcm cells, whereas genes associated with case–control status were mainly identified in the effector cell types (Fig. 3a). Among the 285 disease-associated genes, *CD69*, *PLGRKT*, and *CD226* have also been implicated in recent genome-wide association studies (GWAS) of type 1 diabetes [28], highlighting the potential significance of these genes in type 1 diabetes.

After correction for multiple testing, eight genes were significantly differentially expressed (DE) between cases and controls (FDR < 0.1): *PSME4*, *ZMYM3*, *NTPCR*, *MFHAS1*, *IQGAP2*, *HSD17B10*, *ABCB7*, and *SLC35E4*. These genes were identified as DE within CD69⁺ Tn and Tcm cells (Fig. 3b). Notably, only *PSME4* was significantly downregulated in cases and the while the remaining seven were upregulated in cases (Fig. 3b, c). The direction of expression change between cases and controls was largely similar across the time points (Fig. 3c). Except for *IQGAP2*, the expression differences for the other genes varied across cell types, though these differences did not reach statistical significance (Fig. 3b). Notably, *PSME4* and *NTPCR* were found to be DE in children progressing to type 1 diabetes in earlier studies [6, 7]. Additionally, the promoter region of *SLC35E4* was reported to be hypermethylated in children progressing to type 1 diabetes as compared to matched controls [29]. Further, *MFHAS1* and *IQGAP2* have some known association with diabetes or related disorders [30, 31], whereas the other three genes: *ZMYM3, HSD17B10 and ABCB7* have no known direct association with diabetes.

Given the sensitivity of differential expression results to model assumptions and statistical thresholds, we sought to validate the robustness of our findings by applying Reproducibility-Optimized Test Statistic (ROTS) analysis [23]. We compared gene expression between cases and controls at each time point individually. This approach identified 127, 136, 56, 108, and 122 genes to be associated with case–control status using ROTS at 3, 6, 9, 12, and 18 months, respectively (*p* < 0.001; Additional file 1: Fig. S4a, Additional file 2: Table S15). The majority of these genes were time-point-specific, with only a handful of genes shared across time points (Additional file 1: Fig. S4b). Notably, four of the eight DE genes identified by the LME analysis were also found to be associated with case–control status using ROTS (*p* < 0.001): *IQGAP2* at 3 m, *NTPCR* and *SLC35E4* at 6 m, and *ABCB7* at 18 m. This analysis supports the robustness of our LME-based findings and suggests that these genes may play a role in type 1 diabetes pathogenesis.

To select candidate genes for orthogonal validation by TaqMan assay, we examined the fold change in gene expression between cases and control of these eight genes across different cell types. We reasoned that the gene(s) with consistent differential expression across cell types would be most reliably detected in unsorted bulk samples. *IQGAP2* was the only gene consistently upregulated in cases across all cell types and had the lowest FDR (0.02) (Fig. 3b). Notably, it was upregulated in CD69⁺ naive T cells and showed highest expression in effector Tregs, Th1, and Th2 cells (Additional file 1: Fig. S5a-b). TaqMan analysis confirmed a significant increase in *IQGAP2* expression in all cases, consistent with scRNA-seq data (Additional file 1: Fig. S5c).

### Interferon-mediated signaling pathways along with viral response pathways were altered in children progressing to type 1 diabetes

To investigate the biological pathways affected in children progressing to type 1 diabetes, we performed gene set enrichment analysis (GSEA) on the ranked gene list obtained from the LME analysis (Additional file 2: Table S16). GSEA was selected for its ability to detect coordinated pathway-level changes across the entire transcriptome. Gene sets corresponding to GOBP were used in this analysis. Notably, the "interferon mediated signalling pathway" was significantly enriched in naive and central memory T cells, consistent with previous reports linking interferon responses to type 1 diabetes pathogenesis [32, 33]. In addition, the “response to virus” pathway was enriched across all three naive T cell subsets, central memory T cells, Th17 cells, and naive Tregs. Among the cell type-specific terms, "interferon beta production" was particularly enriched in naive Tregs, while "interleukin-10 production" was enriched in effector Tregs (Fig. 3d). Both IFN-β and IL-10 are crucial for Treg proliferation and their immunosuppressive functions [34, 35].

To further validate these findings, we also performed GSEA on the ranked genes from the ROTS analysis. A total of 191 pathways were enriched of which 34 were shared with the pathways identified from GSEA of the LME analysis (Additional file 1: Fig. S6a, Additional file 2: Table S17). Notably, pathways related to virus response and interferon signalling were shared in both GSEA analyses (Additional file 1: Fig. S6b). The convergence of these results across independent statistical methods strengthens the evidence for dysregulation of antiviral and interferon-related pathways in T cells during early progression to type 1 diabetes.

### SCENIC analysis reveals altered cell-type-specific gene regulatory networks in children progressing to type 1 diabetes

**Fig. 4:**
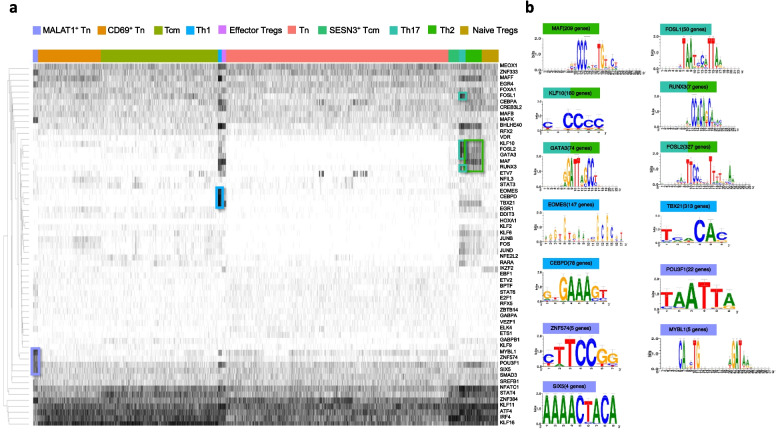
Regulon activity in different CD4^+^ T cell subsets. **a** The binary heatmap shows the activity of 61 top unique regulons identified in each cluster by SCENIC analysis. Each black line indicates that the regulon is active in a given cell. Regulons specifically active in a given cell type have been highlighted on the heatmap. Color code for each cell type has been shown on above the heatmap. **b** Motifs associated with regulons specifically active in a given cell type have been shown. The number of predicted target genes for each regulon have been indicated in the brackets next to the regulons. Color code above the motifs is same as the cell type code in panel A

The scRNA-seq data provide an excellent opportunity for the discovery of cell-type-specific gene regulatory networks (i.e., networks of transcription factor binding at regulatory DNA elements and their target genes), operating under different cellular states. To investigate the gene regulatory networks in different CD4^+^ T cell sub-populations, we performed single cell regulatory network interference and clustering (SCENIC) analysis [25], which identified 245 active regulons across the 10 cell types (Additional file 2: Table S18). A selection of top 10 most active regulons per cluster involved 61 unique regulons (Fig. 4a**,** Additional file 1: Fig. S7). The binary activity of regulons for all the 10 cell types revealed set of regulators as characteristic of cell types (Fig. 4a, b).

For example, regulons for MAF and FOSL1 were the most active in Th2 and Th17 cells, respectively, whereas KLF10, FOSL2 and GATA3 regulons were active in both the cell types. In Th1 cells, EOMES, CEBPD, and TBX21 (encoding T-bet) regulons were the most active (Fig. 4a). Further, MYBL1, ZNF574 and POU3F1 and SIX5 regulons were specifically active in MALAT1^+^ Tn cells (Fig. 4a, b).

Interestingly, seven of eight LME DE genes, except NTPCR, were target of one or more regulons. IQGAP2 was target of target of PRDM1, ELF1, ELF4, EOMES, JUN, KLF6 and MAF. SLC35E4 was a target of JUN. PSME4 was a target of four different regulons ARNTL, CREM, ELF1 and NR3C1. MFHAS1 was target of two regulons MAF and ZNF571. ZMYM3 is target of three regulons: ARID3A, FOXO1, JUND. HSD17B10 was a target of three regulons: KLF13, BCLAF1 and E2F8. ABCB7 was target of three regulons: CHD2, ETS1, and JUN.

**Fig. 5:**
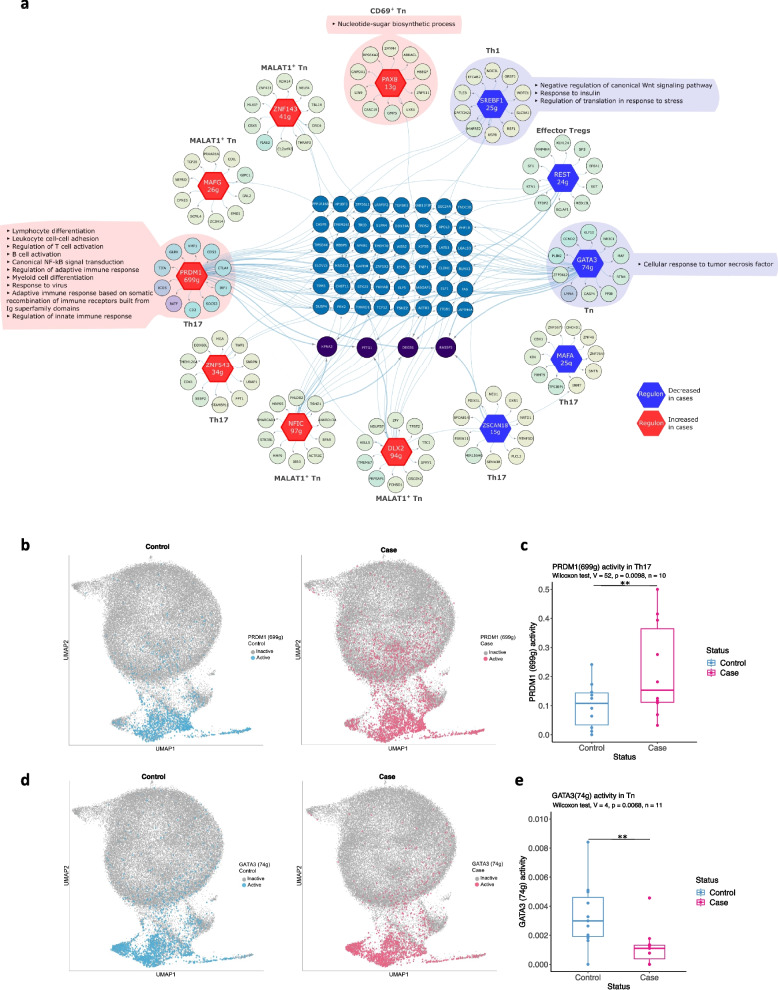
Differentially active regulons between cases and controls. **a** Differentially active regulons, transcription factors (hexagons) and their target genes (circles) have been shown (Wilcoxon signed-rank test: *p* < 0.01). Top 10 unique targets for each regulon are shown along with targets shared by two or three regulons (blue and purple, respectively). Regulons showing increased or decreased activity in cases are indicated with red or blue color of the hexagon, respectively. Pathways enriched among the targets of respective regulons are also shown. **b** UMAP illustrating PRDM1 regulon activity in cases and controls. **c** Box plot shows the activity score of PRDM1 regulons in cases and controls. **d** UMAP illustrating GATA3 regulon activity in cases and controls. **e** Box plot shows the activity score of GATA3 regulons in cases and controls. In box plots, each dot represents an individual child

We next assessed whether regulon activity differed between cases and controls, and found 12 regulons to be differentially active (Wilcoxon signed-rank test, *p*-value < 0.01) in different cell types (Fig. 5a). Four regulons (MAFA, ZNF543, PRDM1, ZSCAN18) showed differential activity between cases and controls in the Th17 cluster and four others (MAFG, ZNF143, NFIC, DLX2) were differentially active in MALAT1^+^ Tn cells. GATA3, PAX8, SREBF1, and REST regulons showed differential activity in Tn cells, CD69^+^ Tn cells, Th1, and effector Tregs, respectively. Of the 12 differentially active regulons, seven regulons (PRDM1, PAX8, DLX2, NFIC, ZNF543, MAFG, and ZNF143) were more active in cases, whereas five regulons (REST, MAFA, SREBF1, ZSCAN18, and GATA3) were less active in cases (Fig. 5a). Each of the regulon TFs regulated unique as well as shared target genes across different T cell clusters. Among the regulons whose activity was up- and downregulated, the highest number of shared target genes were observed for PRDM1 and GATA3, respectively. The regulon activity of PRDM1 was upregulated in cases in Th17 cells (Fig. 5b, c), whereas GATA3 regulon activity was downregulated in cases in Tn cells (Fig. 5d, e). These findings suggest that dysregulated activity of key transcriptional regulators, particularly PRDM1 and GATA3, may contribute to the altered gene expression landscapes in specific CD4⁺ Tcell subsets during early type 1 diabetes development.

To further explore the biological functions associated with the differentially active regulons, we performed pathways enrichment analysis for GOBP for each of the differentially active regulons (Additional file 2: Table S19). PRDM1 targets had highest number of pathways enriched, likely due to its large regulon size, including pathways related to regulation of innate and adaptive immune response, lymphocyte differentiation, response to virus and canonical NF-κB signal transduction (Fig. 5a). SREBF1 targets had enrichment of three pathways: negative regulation of canonical Wnt signalling, response to insulin and regulation of translation in response to stress. PAX8 and GATA3 targets had the enrichment of nucleotide sugar biosynthetic processes and cellular response to tumour necrosis factor, respectively (Fig. 5a).

Finally, we compared these SCENIC-derived pathways to those identified through GSEA of the LME-ranked gene list (Additional file 2: Table S14 and S19). As shown in Additional file 1: Fig. S8, 10 ten pathways were common between the two analyses. These include pathways of T cell activation and differentiation and response to viruses, providing further support for the role of immune response to viruses in the pathogenesis of type 1 diabetes.

## Discussion

In this study, we utilized scRNA-seq to investigate gene expression changes in CD4^+^ T cells early during the development of type 1 diabetes analysing longitudinal samples from a prospective cohort of early progressors who developed the disease before the age of 5 years. Analysing over 99,000 single cells, we could identify 10 subtypes of CD4^+^ T cells, including clusters of naive, central memory, effector and regulatory T cells. One of the key findings of our study is the identification of 785 and 285 genes whose expression was associated with age or disease status, respectively, within these CD4^+^ T cell subsets. Notably, these gene expression changes were not uniformly observed across all CD4⁺ T cells, underscoring the importance of cellular heterogeneity. Additionally, the cell type proportions were similar between cases and controls. Importantly, we identified eight genes, *PSME4*, *ZMYM3*, *NTPCR*, *MFHAS1*, *IQGAP2*, *HSD17B10*, *ABCB7*, and *SLC35E4*, as DE between cases and controls. Interestingly, pathways related to viral response and interferon signalling were found to be altered in cases. Furthermore, 12 regulons, including PRDM1 regulon which was more active in Th17 cells of cases, were found to be differentially regulated between cases and controls.

Among the eight DE genes, *PSME4* and *NTPCR* were shown to be DE in children progressing to type 1 diabetes, in earlier studies [6, 7], and the promoter region of *SLC35E4* was hypermethylated in children progressing to the disease before seroconversion as we reported earlier [29]. PSME4 was the only downregulated gene in cases. Reduced PSME4 results in an active immunoproteasome, leading to a pro-inflammatory environment in non-small-cell lung carcinoma [36]. Thus, its downregulation may contribute to increased inflammation in type 1 diabetes. Indeed, the proteasome-mediated degradation pathway was reported to be altered in children progressing to beta-cell autoimmunity [37]. Additionally, IQGAP was consistently upregulated in cases across cell types and was validated by TaqMan assay, confirming the higher expression of the gene in cases in this cohort. IQGAP2 interacts with glycogen synthesis regulators and affects the phosphorylation of components of insulin pathway in liver [31]. Furthermore, in mice, *Iqgap2* deletion resulted in increased plasma insulin levels [38].

Among the genes associated with disease status (LME: *p* < 0.001), some genes have previously been reported to be increased in children progressing to type 1 diabetes. For instance, *HLA-F*, upregulated in MALAT1^+^ Tn cells, was reported to be upregulated in two earlier bulk transcriptomics studies [7, 39]. Similarly, *GZMA* and *CST7*, which were upregulated in cases in Naive Tregs and Th17 cells, respectively, were also upregulated in cases in three earlier bulk transcriptomics studies [4, 6, 7]. *RGS14* and *SUSD3*, which were upregulated in our study were also reported earlier in bulk RNA-seq data of CD4^+^ T cells from children progressing to the disease [7]. While *HLA-F* polymorphism has been associated with autoimmune diseases, including multiple sclerosis and rheumatoid arthritis, they have not yet been linked to type 1 diabetes (GWAS Catalogue: accessed on Mar 21, 2024). Granzyme A, encoded by *GZMA,* is a classical proapoptotic mediator of immune-mediated killing secreted mainly by effector T cells such as Th1 but also by Tregs. Interestingly, deleting *Gzma,* accelerated the onset of autoimmune diabetes in nonobese diabetic (NOD) mice [40], suggesting that this effect was due to impaired regulatory T cell (Treg) function, as *Gzma* is a key effector molecule in Tregs [41]. In our data, more cells expressed granzyme A in the effector T cell clusters than the regulatory T cell cluster, but the difference between cases and controls was significant only in naive Tregs. The precise mechanism of action of these gene products on the pathogenesis of type 1 diabetes remains to be determined.

Epigenetic changes, including DNA methylation and histone modifications, regulate gene expression in health and disease. In our data, DNMT1 that maintains DNA methylation post-replication, was upregulated in SESN3^+^ Tcm cells, and TET1, that actively demethylates DNA was upregulated in naive Tregs. Further, the DNMT1-associated gene, *DMAP1* was upregulated in Th2 cells. Alterations in histone acetylation have been associated with diabetic retinopathy, nephropathy, and encephalopathy [42–45]. We found *HDAC7,* which is responsible for deacetylation of core histone, was upregulated in cases in Tn cells. Further, *PSME4,* the gene that encodes a proteosome activator that specifically promotes degradation of acetylated histones [46], was downregulated in cases in multiple cell types, including Tn and Tcm cells, naive Tregs, and Th2 cells (Fig. 3B, C). Furthermore, it was downregulated in our earlier scRNA-seq study of children progressing to type 1 diabetes [7]. Intriguingly, *ZMYM3*, which is a part of histone deacetylase-containing multiprotein complexes, was upregulated in cases in CD69^+^ Tn cells and Tcm cells (Fig. 3B, C). These observations suggest an interesting link between epigenetic changes and type 1 diabetes.

The enrichment of interferon response pathways in our data corroborates previous findings from bulk sample analyses conducted by our group [33] and others [32]. Another pathway consistently found to be upregulated in cases in different cell types was viral response and related pathways, potentially providing further evidence supporting the existing links between viruses and type 1 diabetes [47, 48].

SCENIC analysis provided further insights into the gene regulatory networks operating within different CD4^+^ T cell sub-populations. Importantly, we identified 12 regulons with differential activity between cases and controls, highlighting potential regulatory mechanisms that may contribute to type 1 diabetes development. The increase in PRDM1, also known as BLIMP1, regulon activity in Th17 cells in cases is particularly noteworthy. Prdm1 regulates T cell activation [49] and prevents inflammation by repressing *Il17a* locus [50] and upregulating FOXP3 expression [51]. Furthermore, T cell-specific deletion of Blimp1 led to increased Th1 and Th17 cell polarization in vivo [52]. Additionally, Blimp1 overexpression in T cells significantly reduced insulitis and diabetes in NOD mice [52]. These studies suggest that PDRM1 contributes to reducing the inflammation by modulating the gene expression program of CD4^+^ T cells. The increased PRDM1 regulon activity observed in Th17 cells of children progressing to the disease may reflect a compensatory regulatory response during the early stages of disease. However, the persistence of autoimmunity despite this upregulation suggests that such mechanisms are either insufficient or overridden by other pro-inflammatory signals.

PRDM1 has a complex relationship with RORγt, the lineage defining factor for Th17. In mice, Prdm1 negatively regulates Rorc, as conditional deletion of Prdm1 in CD4⁺ T cells results in increased Rorc and Il17a expression [53] and attenuates autoimmune diabetes in NOD mice by suppressing both Th1 and Th17 cells [52]. In humans, using time-series data generated in our laboratory, Acerbi et al. identified PRDM1 as a negative regulator of Th17 differentiation: siRNA-mediated silencing of PRDM1 in CD4⁺ T cells led to upregulation of IL17A but did not alter RORC expression [54]. Conversely, pharmacological inhibition of RORγt increased Blimp1 expression [55]. Together, these findings suggest that PRDM1 and RORC may negatively regulate each other in Th17 cells. On the other hand, Jain et al. observed that peripheral deletion of Blimp1 reduced Th17 activation and experimental autoimmune encephalomyelitis and that Blimp1-mediated functions in Th17 cells are dependent on RORC [56]. Together, these observations highlight the complex and context-dependent interplay between PRDM1 and RORC in regulating human and murine Th17 cell differentiation and function.

In the cohort of our present study, the children progressed to seroconversion before the age of 2 years and developed type 1 diabetes before the age of 5 years. This homogeneous cohort of fast progressors is a critical aspect of our sample set because type 1 diabetes is increasingly recognized as a heterogeneous disease, characterized by variability in the age of diagnosis and the type of autoantibodies that first appear during beta cell autoimmunity [57]. Our recent study provides additional evidence for distinct cellular immunity associated with the first-appearing autoantibody in children who progress to the disease [58]. Therefore, to find features characteristic for late-onset or slow-progressing forms of type 1 diabetes having cohorts homogeneous for these characteristics would be important for future studies.

The cohort size was relatively small, and the conclusions should be interpreted in this context; nevertheless, the findings provide important insights and a foundation for future studies. In this study, we focused our analysis on the CD4⁺ T cell compartment, which enabled us to explore in greater depth the dysregulation of various CD4⁺ T cell subsets during disease progression. However, this focus represents an important limitation, as it excludes CD8⁺ T cells and other immune cell types from the analysis. Given the established roles of CD8⁺ T cells, B cells, and innate immune cells in type 1 diabetes, future studies incorporating these populations will provide valuable perspectives. Future single-cell studies with a longitudinal design, conducted in larger cohorts and integrating data from multiple immune cell types as well as pancreatic islets, would be essential to gain a more comprehensive understanding of disease mechanisms.

## Conclusions

Our study provides a comprehensive single-cell transcriptomic analysis of CD4⁺ T cells during the early, preclinical stages of type 1 diabetes in a homogeneous cohort of fast-progressing children. We identified cell-type-specific changes in gene expression and gene regulatory networks, including differential expression of key genes and altered interferon and viral response pathways. Notably, differential regulon activity points to disrupted immune regulation in children progressing to the disease. These findings underscore the importance of early immune dysregulation and cellular heterogeneity within CD4^+^ T cell compartment in type 1 diabetes pathogenesis, offering novel insights into aberrant temporal and cell-type–specific immune regulation driving disease progression. Future longitudinal single-cell studies in larger and more diverse cohorts will be crucial to further delineate disease subtypes and mechanisms.

## Supplementary Information

Supplementary Material 1. Figure S1: Quality metrices for scRNA-seq data. Figure S2: Cell recovery and clustering in each case-control pair. Figure S3: CD69^+^ T cells fraction in naive and memory CD4^+^ T cells in children. Figure S4: Gene expression changes between cases and controls at different time points as identified by ROTS analysis. Figure S5: IQGAP2 expression in scRNA-seq and TaqMan data. Figure S6: Pathway overlap between LME and ROTS analyses. Figure S7: Regulon specificity score for each CD4^+^ cell subtypes. Figure S8. Comparison of enriched pathway between GSEA of LME dataset and GO enrichment of regulon targets.

Supplementary Material 2. Table S1: Metadata table. Table S2: Cell hashing antibody for cell multiplexing used for scRNA-seq. Table S3. Sample staining and pooling scheme used for scRNA-seq Table S4. Marker gene list for all ten clusters annotated. Table S5 – S14: Linear Mixed Effext (LME) modelling analysis for genes affected by status (Case vs Control), age, sex and interaction of status and age in all ten clusters annotated. Table S15: ROTS analysis for genes affected by status at five timepoints. Table S16: GSEA results on T1D status associated genes from LME analysis across seven cell types. Table S17: GSEA results on T1D status associated genes from ROTS analysis across cell types identified five timepoints. Table S18: Regulon specificity score for all 245 regulons identified across ten cell types. Table S19: GO over-representaion results for differentially expressed regulons together with their target genes.

## Data Availability

The raw epidemiological and sequencing data are protected and not available due to data privacy laws. The processed data underlying each figure and table are provided in the Supplementary Information. Processed single cell count expression matrix has been deposited to ArrayExpress at EBI and can be accessed with accession code: E-MTAB-15832. Custom codes used in this analysis has been deposited at Zenodo repository (10.5281/zenodo.17338297). The metadata sisi provided as Additional file 2: Table S1. All analysis tools and packages used here were published and are cited in the main text or methods section.

## Abbreviations

FDR: False discovery rate
GOBP: Gene ontology biological processes
GSEA: Gene set enrichment analysis
GWAS: Genome wide association studies
HLA: Human leukocyte antigen
IFN-β: Interferon beta
IL-10: Interleukin 10
LME: Linear mixed effect
NOD: Non obese diabetic
PBMC: Peripheral blood mononuclear cells
ROTS: Reproducibility-optimized test statistic
SCENIC: Single cell regulatory network inference and clustering
scRNA-seq: Single cell RNA sequencing
