# Supplementary material for: Single-cell RNA-seq analysis of longitudinal CD4+ T cell samples reveals cell-type-specific changes during early stages of type 1 diabetes
Source: Genome Med. 2025 Dec 29;17:154. doi: 10.1186/s13073-025-01574-x (PMC12751217; doi:10.1186/s13073-025-01574-x)
Supplement: Supplementary file 1 — Supplementary Material 1. Figure S1: Quality metrices for scRNA-seq data. Figure S2: Cell recovery and clustering in each case-control pair. Figure S3: CD69+ T cells fraction in naive and memory CD4+ T cells in children. Figure S4: Gene expression changes between cases and controls at different time points as identified by ROTS analysis. Figure S5: IQGAP2 expression in scRNA-seq and TaqMan data. Figure S6: Pathway overlap between LME and ROTS analyses. Figure S7: Regulon specificity score for each CD4+ cell subtypes. Figure S8. Comparison of enriched pathway between GSEA of LME dataset and GO enrichment of regulon targets. [file 13073_2025_1574_MOESM1_ESM.docx]

**Figure S1. Quality metrices for scRNA-seq data** Each box represents one sample pool processed together for a Chromium run. Heatmap indicates expression of individual hashtag-labelled multiplexed sample. nFeature_RNA and nFeature_count in the violin plots on the right show number unique RNA in each cell and number of RNAs identified in a cells. Percent_mt shows the mitochondrial RNA percent in each cell.

**Figure S2: Number of recovered cells and number of clusters at clustering resolution 1.2** (**a-b**) The bar charts number of recovered cells that passed quality control and median number of genes per cell for each case-control child pair processed together. (**c**) Number of clusters before merging and annotations. (**d**) UMAPs showing the contribution of cells to different clusters by all 11 pairs.

**Figure S3: CD69^+^ T cells fraction in naive and memory CD4^+^ T cells in children** (Reanalysis of PBMC scRNA-seq data from GSE206295) (**a-c**) UMAP visualization of clusters (a), violin (b) and bar plot (c) showing the expression of CD69 in naive and memory CD4^+^ T cells from child 1. (**d-f**) Similar visualization as in a-c for child 2 and for child 3 (**g-i**).

**Figure S4: Gene expression changes between cases and controls at different time points as identified by ROTS analysis** (**a**) Number of genes associated with case-control status (Status) (p<0.001). Red and blue bars show the number of upregulated and downregulated genes in cases, respectively. (**b**) Heatmaps showing the overlap of genes associated with status as identified by ROTS and LME analyses.

**Figure S5. IQGAP2 expression in scRNA-seq and TaqMan data** (**a-b)** visualize IQGAP2 expression in different cell types in dot and violin plots, respectively. (**c**) shows IQGAP2 expression in TaqMan data. The significance was determined using paired t-test. **: p<0.01

**Figure S6. Pathway overlap between LME and ROTS analyses** (**a**) The Venn diagram shows the overlap pf enriched pathways (GSEA) on the ranked list of genes from LME and ROTS analyses and (**b**) shows 34 common pathways.

**Figure S7: Regulon specificity score for each CD4^+^ cell subtypes** The top ten regulons in each cell type are highlighted in red and labelled on the plot. The specificity score is shown on the y axis.

**Figure S8. Comparison of enriched pathway between GSEA of LME dataset and GO enrichment of regulon targets** The Venn diagram shows the overlap pf enriched pathways (GSEA) on the ranked list of genes from LME and the targets of regulons. Common pathways are highlighted.
